# Inhibitory effects of evodiamine on human osteosarcoma cell proliferation and apoptosis

**DOI:** 10.3892/ol.2014.2791

**Published:** 2014-12-11

**Authors:** XIAODONG BAI, HAI MENG, LIFENG MA, AI GUO

**Affiliations:** Department of Orthopedics, Beijing Friendship Hospital, Capital Medical University, Xicheng, Beijing 100050, P.R. China

**Keywords:** evodiamine, osteosarcoma cells, proliferation, apoptosis

## Abstract

Osteosarcoma is a primary malignancy of bone, which is characterized by the proliferation of malignant mesenchymal cells, particularly in children and adolescents. Evodiamine is extracted from a variety of traditional Chinese medicines, which has been reported to induce apoptosis in certain tumors, including cervical, prostate and breast cancer, however, its effect on oestosarcoma cells remains unclear. The aim of the present study was to investigate the effect of evodiamine on osteosarcoma cell proliferation and apoptosis, and explore the associated underlying molecular mechanism. A Cell Counting Kit 8 assay was performed to detect the effects of evodiamine on the proliferation of human osteosarcoma U2OS cells. Annexin V-fluorescein isothiocyanate/propidium iodide staining was performed to analyze the apoptotic rate of the cells. The effect of evodiamine on the protein expression levels of B-cell lymphoma-2 (Bcl-2), Bcl-2-associated X protein (Bax), caspase-3 and survivin were detected by performing western blot analysis. Evodiamine inhibited the growth of human osteosarcoma U2OS cells by inhibiting cell proliferation and inducing cell apoptosis. Western blotting demonstrated that evodiamine downregulated the expression of Bcl-2, caspase-3 and survivin, and upregulated the expression of Bax in human osteosarcoma cells. Evodiamine effectively inhibited proliferation and induced apoptosis of osteosarcoma cells in a dose-dependent manner via downregulation of Bcl-2, caspase-3 and survivin protein expression levels and upregulation of Bax protein expression levels.

## Introduction

Osteosarcoma is the most commonly diagnosed primary malignancy of bone, particularly among children and adolescents (1,2). It is characterized by the proliferation of malignant mesenchymal cells that are capable of producing osteoid or immature bone (3), and is associated with high morbidity, early metastasis and mortality (4–6). Survival of osteosarcoma is poor despite the aggressive use of surgery, chemotherapy, and/or radiotherapy (7). Furthermore, surgery cannot halt the metastasis of the tumor and chemotherapy is restricted by the development of resistance and various side effects. Currently, traditional Chinese medicine (TCM) is commonly administered to cancer patients as an adjunct to conventional therapies to improve the quality of life by alleviating symptoms and side effects (8). The active gradients of TCM have been demonstrated to inhibit tumor cell proliferation and induce tumor cell apoptosis (9,10).

Evodiamine is a natural alkaloid compound extracted from a variety of TCMs, such as *Evodia rutaecarpa* (Juss.) Benth., *E. rutaecarpa* (Juss.) Benth. var. officinalis (Dode) Huang and *E. rutaecarpa* (Juss.) Benth. var. bodinieri (Dode) Huang. Vasodilatation (11), anti-inflammation (12), analgesia (13) and anti-tumor (14) effects are the major efficacies of evodiamine. Additionally, evodiamine has been shown to induce the apoptosis of various tumors, such as cervical (15), prostatic (16,17) and breast cancer (18), as well as melanoma (19) and leukemia (20), via the inhibition of proliferation, blockade of the cell cycle, induction of apoptosis and suppression of metastasis. However, to the best of our knowledge, no studies been conducted that focus on the effects of evodiamine in osteosarcoma.

In the present study, human osteosarcoma U2OS cells were cultured with different concentrations of evodiamine to explore its effect on U2OS cell proliferation and apoptosis. Additionally, the expression levels of apoptosis-associated proteins were determined to understand the underlying mechanism.

## Materials and methods

### Reagents

Evodiamine was purchased from the National Institutes for Food and Drug Control (Beijing, China), and dimethyl sulfoxide, RPMI-1640 medium and fetal bovine serum (FBS) were purchased for cell culture from Gibco-BRL (Carlsbad, CA, USA). An annexin V-fluorescein isothiocyanate/propidium iodide (FITC/PI) kit was purchased from Sigma-Aldrich (St. Louis, MO, USA) for cell apoptosis detection, and a Cell Counting Kit 8 (CCK-8) was purchased from Beyotime Institute of Biotechnology (Haimen, China) for the cell viability assay. Furthermore, for western blot analysis, monoclonal mouse anti-human B-cell lymphoma (Bcl-2; 1:1,000; cat. no. sc-130307) and polyclonal mouse anti-human Bcl-2-associated X protein (Bax; 1:1,000; cat. no. sc-20067) antibodies, and polyclonal peroxidase-conjugated goat anti-mouse (1:1,000; cat. no. sc-2354) antibodies were purchased from Santa Cruz Biotechnology, Inc. (Dallas, TX, USA). Monoclonal mouse-anti-human caspase-3 (1:1,000; cat. no. 9668), mouse anti-human β-actin (1:1,000; cat. no. 3700) and mouse anti-human survivin (1:1,000; cat. no. 2802) antibodies were purchased from Cell Signaling Technology (Danvers, MA, USA). The luminol-enhanced chemiluminescence kit was obtained from GE Healthcare (Chalfont, UK).

### Cell line and culture conditions

Human osteosarcoma U2OS cells were purchased from the Cell Bank of the Chinese Academy of Sciences (Shanghai, China) and grown in RPMI-1640 medium with 10% (v/v) FBS, 100 U/ml streptomycin and 100 U/ml penicillin. The cell cultures were maintained in a 37°C incubator with a humidified atmosphere of 5% CO_2_. U2OS cells were harvested in the logarithmic growth phase and used in following experiments.

### CCK-8 assay

The viability of the U2OS cells was assessed using a CCK-8 assay. The U2OS cells (1×10^4^/ml) were plated on a 96-well plate at 100 μl/well and incubated overnight. A total of 24 wells containing cultured cells of the 96-well plate were then divided into four groups (n=6 wells in each group): control group [treated with 0.1% DMSO (v/v)] and three evodiamine groups (treated with 0.5, 2.5 and 12.5 μg/ml, respectively). After culturing with and without evodiamine for 48 h, 10 μl CCK-8 solution was added to each well, according to the manufacturer’s instructions. After 2 h incubation at 37°C, the optical density value was measured at a wavelength of 450 nm. The following formula was used to calculate the cell survival rate: Cell survival rate (%) = 1 − {[(control well) − (evodiamine well)] / (control well)} × 100%.

### Annexin V-FITC/PI flow cytometry analysis

U2OS cells (1×10^6^/ml) were plated on a 96-well plate at 2,000 μl/well and incubated overnight. A total of 24 wells containing cultured cells of the 96-well plate were then divided into four groups (n=6 wells in each group): control group [treated with 0.1% DMSO (v/v)] and three evodiamine groups (treated with 0.5, 2.5 and 12.5 μg/ml, respectively). After culturing with and without evodiamine for 48 h, the cells were harvested, and the percentages of apoptotic or necrotic cells were determined using the Annexin V-FITC/PI Apoptosis Detection kit, according to the manufacturer’s instructions, in a flow cytometer (FACSCalibur™; BD Biosciences, Franklin Lakes, NJ, USA).

### Western blot analysis

The expression levels of cellular proteins were evaluated by performing western blot analysis. U2OS cells (1×10^6^/ml) were plated on a 96-well plate at 2,000 *μ*l/well and incubated overnight. A total of 24 wells containing cultured cells of the 96-well plate were then divided into four groups (n=6 wells in each group): control group [treated with 0.1% DMSO (v/v)] and three evodiamine groups (treated with 0.5, 2.5 and 12.5 μg/ml, respectively). Following treatment for 12 h, total proteins were extracted and the protein concentrations were determined using a bicinchoninic acid protein assay. Equal amounts of protein from each sample were separated by 12% SDS-PAGE. Following electrophoresis, the proteins were electroblotted onto polyvinylidene difluoride membranes for 1 h at room temperature. Subsequently, the membranes were individually incubated with the following primary antibodies (dilution, 1:1,000) overnight at 4°C: Mouse anti-human Bcl-2 monoclonal antibody, mouse anti-human Bax polyclonal antibody, mouse anti-human caspase-3 and survivin monoclonal antibody. The membranes were washed three times and incubated with the secondary peroxidase-conjugated antibody (dilution, 1:1,000) for 1 h at room temperature. Following incubation, the membranes were washed and the peroxidase activity was visualized on X-ray film using the luminol-enhanced chemiluminescence kit (GE Healthcare), according to the manufacturer’s instructions. β-actin was used as a reference for normalization. The protein bands were quantified using ImageJ software (http://rsb.info.nih.gov/ij/) (National Institutes of Health, Bethesda, MD, USA).

### Statistical analysis

The data are expressed as the mean ± standard deviation. Statistical correlation of data was checked for significance by analysis of variance and Student’s t-test. P<0.05 was considered to indicate a statistically significant difference. These analyses were performed using SPSS software (version 11; SPSS, Inc., Chicago, IL, USA).

## Results

### Evodiamine suppresses proliferation in human osteosarcoma U2OS cells

**Figure 1:**
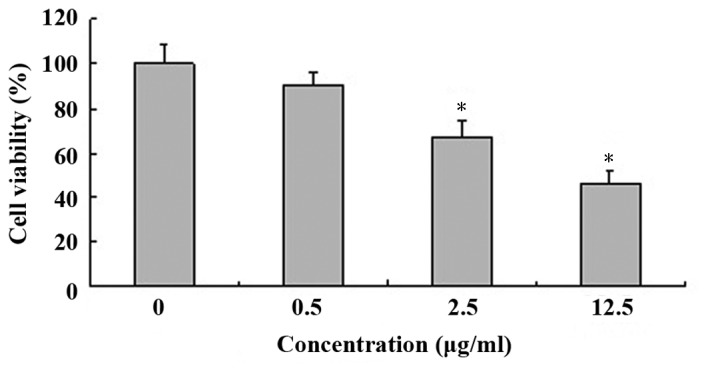
Proliferative inhibition effects of evodiamine on human osteosarcoma U2OS cells (^*^P<0.05, vs. the control group).

CCK-8 assays were performed to detect the impact of evodiamine on the proliferation of U2OS cells. The U2OS cell line was incubated with 0, 0.5, 2.5 and 12.5 μg/ml evodiamine. After 48 h incubation, the U2OS cells treated with 0.5, 2.5 and 12.5 μg/ml evodiamine exhibited reduced levels of cell viability, to 89.90±6.12, 66.65±8.01 and 46.22±6.23%, respectively, compared with the control group (Fig. 1). These results indicated that evodiamine treatment induced cell growth inhibition in a concentration-dependent manner in the U2OS human osteosarcoma cell line. The cells treated with 2.5 (P<0.05) and 12.5 μg/ml (P<0.05) evodiamine exhibited a statistically significant reduced level of cell viability compared with the control group.

### Evodiamine induces apoptosis in human osteosarcoma U2OS cells

**Figure 2:**
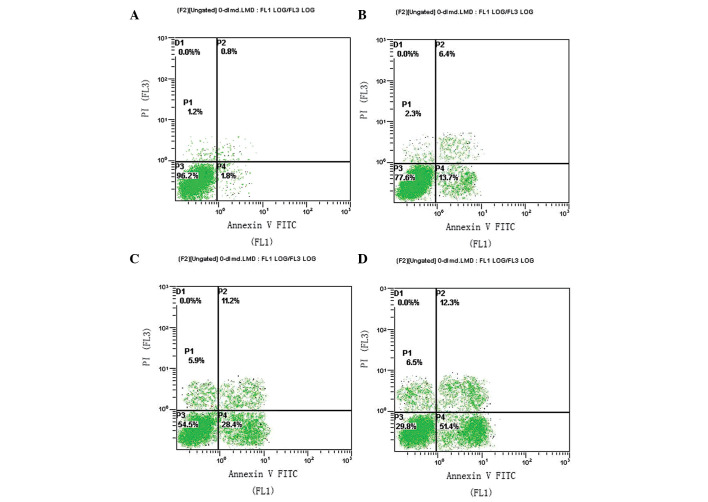
Effect of evodiamine on the apoptosis of U2OS cells using flow cytometry. (A) Control group; (B) 0.5 μg/ml evodiamine; (C) 2.5 μg/ml evodiamine; and (D) 12.5 μg/ml evodiamine.

U2OS cells were incubated with 0, 0.5, 2.5 and 12.5 μg/ml evodiamine for 48 h and were analyzed by flow cytometry. The proportions of early apoptotic cells were determined as 1.8, 13.7, 28.4 and 51.4%, respectively (Fig. 2). These results indicated that evodiamine treatment induced cell apoptosis in a concentration-dependent manner in the U2OS human osteosarcoma cell line.

### Effect of evodiamine on the expression levels of Bcl-2 and Bax protein

**Figure 3:**
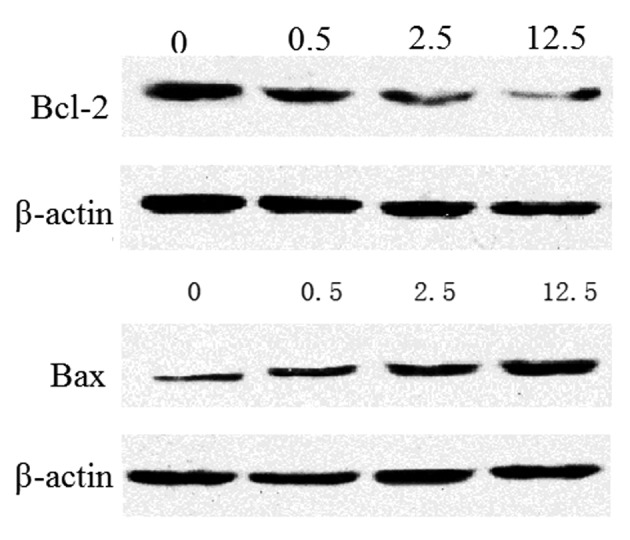
Protein expression levels of Bcl-2 and Bax following treatment with increasing concentrations of evodiamine for 12 h. 0, control group; 0.5, 0.5 μg/ml evodiamine; 2.5, 2.5 μg/ml evodiamine; 12.5, 12.5 μg/ml evodiamine.

To determine the molecular mechanism by which evodiamine induces the apoptosis of U2OS cells, the protein expression levels of Bcl-2 (an inhibitor of apoptosis) (21) and Bax (a pro-apoptotic member of the Bcl-2 family) (22) were assessed by performing western blot analysis. As indicated in Fig. 3, quantitative analysis revealed that the protein expression levels of Bcl-2 significantly decreased (P<0.05) and the protein expression levels of Bax significantly increased (P<0.05) following treatment with evodiamine for 12 h, compared with the control group. Therefore, evodiamine may induce apoptosis of U2OS cells through the mitochondrial pathway.

### Evodiamine decreases the expression levels of caspase-3 in U2OS cells

**Figure 4:**
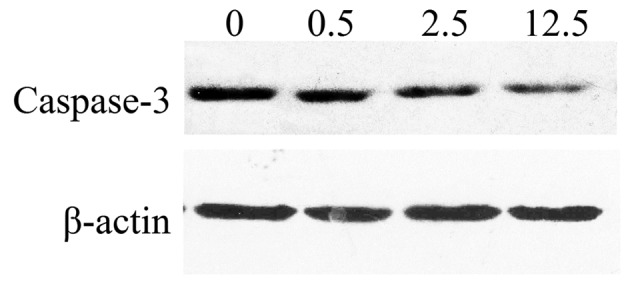
Protein expression levels of caspase-3 following treatment with increasing concentrations of evodiamine for 12 h. 0, control group; 0.5, 0.5 μg/ml evodiamine; 2.5, 2.5 μg/ml evodiamine; 12.5, 12.5 μg/ml evodiamine.

The expression levels of caspase-3 (a pro-apoptotic protein) was downregulated in a concentration-dependent manner as the concentration of evodiamine increased (Fig. 4). The results indicated that the apoptosis induced by evodiamine may involve the caspase cascade.

### Evodiamine decreases the expression levels of survivin in U2OS cells

**Figure 5:**
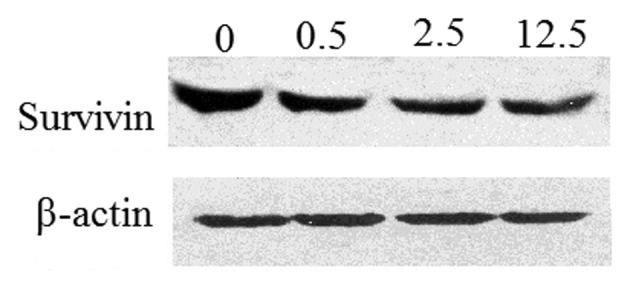
Protein expression levels of survivin following treatment with increasing concentrations of evodiamine for 12 h. 0, control group; 0.5, 0.5 μg/ml evodiamine; 2.5, 2.5 μg/ml evodiamine; 12.5, 12.5 μg/ml evodiamine.

Survivin, an anti-apoptotic protein, exerts an important role in the development of tumors. The expression levels of survivin were downregulated in a concentration-dependent manner as the concentration of evodiamine was increased (Fig. 5). The results indicated that survivin may be a target of the apoptosis pathway induced by evodiamine.

## Discussion

Previous studies have demonstrated the apoptosis-inducing and chemotherapy resistance-reversing effects of TCMs and their active ingredients (8–10). Evodiamine is one of the main constituents of *Evodiae fructus*, and exhibits antitumor and antiproliferative properties (16,23). In the present study, experimental data demonstrated that evodiamine significantly inhibits the proliferation and induces the apoptosis of U2OS cells in a dose-dependent manner. Following 48 h co-culturing with 12.5 μg/ml evodiamine, the cell viability was reduced to 46.22±6.23% and the proportion of early apoptotic cells was 51.4%, which indicates that evodiamine may efficiently inhibit the proliferation of U2OS cells. Tumorigenesis is closely associated with the loss of control of cell proliferation and diminished apoptosis, thus, evodiamine administration may exert a curative effect on osteosarcoma patients.

Programmed cell death, or apoptosis, is important for the development and homeostasis of the majority of tissue types (24). Apoptosis is regulated by various factors and signaling pathways, such as the endoplasmic reticulum pathway, the mitochondrial pathway and the death ligand pathway (25). The mitochondrial pathway is activated in response to the activation of the anti-apoptotic protein Bcl-2 and the pro-apoptotic protein Bax of the Bcl-2 family, which promote the secretion of cytochrome *c* (24) and activate caspase-3 and -9 in the downstream signaling pathways of Bcl-2 and Bax (26–28). However, Bcl-2 indirectly inhibits caspase-3 activation in a variety of pro-apoptotic conditions (29). Bcl-2 may prevent the accumulation of cytochrome *c*, subsequently preserving capase-3 in the inactive zymogen state, which leads to the inhibition of the apoptotic cascade (30). A previous study demonstrated that reduced Bcl-2 expression levels caused by evodiamine administration resulted in increased mitochondrial cytochrome *c* release, and an increased ratio of Bax/Bcl-2 expression was closely associated with evodiamine-induced apoptosis (14). Survivin, an anti-apoptotic protein, may facilitate apoptosis-resistance in specific cells, and is an important target in current antitumor research (31–32). Survivin specifically binds to members of the caspase family of proteins and inhibits the activity of caspase-3 to block apoptosis. Furthermore, survivin expression is positively associated with the expression levels of Bcl-2, showing a synergistic effect, and is negatively associated with the expression levels of Bax, demonstrating an antagonistic effect (33–36).

Western blot analysis was performed to detect the expression levels of apoptosis-associated proteins Bcl-2, Bax, caspase-3 and survivin, and it was identified that the ratio of Bax/Bcl-2 increased with increasing concentrations of evodiamine. The differential expression of caspase-3 verified that evodiamine may induce apoptosis of U2OS tumor cells via the mitochondrial pathway. In addition, survivin is an independent index of osteosarcoma prognosis (31,37,38); in the present study, the expression levels of survivin decreased with increasing concentrations of evodiamine. Thus, survivin served as a target for the regulation of evodiamine-induced apoptosis in osteosarcoma, and was associated with Bcl-2, Bax and caspase-3 expression levels.

In conclusion, the present study identified that the mitochondrial pathway and induced survivin expression may be mechanisms by which evodiamine inhibits proliferation and induces apoptosis in osteosarcoma cells. Therefore, evodiamine may be used as an adjuvant agent to chemotherapeutics in the treatment of osteosarcoma. However, additional studies are required to explore the toxicity of evodiamine *in vivo,* as well as its tolerance and pharmacokinetic characteristics.
